# Supplementary material for: How to Distinguish Marfan Syndrome from Marfanoid Habitus in a Physical Examination—Comparison of External Features in Patients with Marfan Syndrome and Marfanoid Habitus
Source: Int J Environ Res Public Health. 2022 Jan 11;19(2):772. doi: 10.3390/ijerph19020772 (PMC8775541; doi:10.3390/ijerph19020772)
Supplement: Supplementary file 1 [file ijerph-19-00772-s001.zip › ijerph-1475338-supplementary.pdf]

**Child and adult population (n=277)**	**Univariate logistic regression**			**Multivariate logistic regression**		
	**p**	**HR**	**95%CI**	**p**	**HR**	**95%CI**
USLS <0.85	0.557	0.72	0.23-2.19	-	-	-
ASHR >1.05	<0.001	4.44	2.36-8.36	0.035	3.48	1.09-11.11
thumb sign	0.018	1.92	1.11-3.29	0.643	1.32	0.41-4.20
wrist sign	0.023	1.79	1.09-2.95	0.939	0.96	0.32-2.84
pectus carinatum	<0.001	4.86	2.68-8.83	0.048	3.00	1.01-8.92
pectus excavatum	0.899	1.04	0.59-1.82	-	-	-
scoliosis	0.019	2.17	1.14-4.14	0.557	1.40	0.45-4.35
assymetry of the chest	<0.001	3.41	1.91-6.08	0.410	1.58	0.53-4.65
joint laxity	0.662	1.12	0.67-1.90	-	-	-
joint hyperextension	0.207	0.73	0.44-1.19	-	-	-
reduced elbow extension	0.002	5.94	1.88-18.78	0.816	1.27	0.17-9.60
dolichocephaly	0.761	1.09	0.64-1.86	-	-	-
malar hypoplasia	0.286	1.31	0.80-2.15	-	-	-
enophthalmia	0.051	1.69	1.00-2.87	0.381	1.53	0.59-3.93
micrognation	0.015	1.87	1.13-3.09	0.336	0.62	0.23-1.64
retrognathia	0.130	1.53	0.88-2.64	-	-	-
downslanting palpebral fissures	<0.001	3.12	1.84-5.28	0.167	1.94	0.76-4.98
gothic palate	<0.001	6.44	3.73-11.14	0.221	1.79	0.71-4.53
dental crowding	0.001	2.38	1.42-3.98	0.403	1.52	0.57-4.06
stretch marks	<0.001	3.40	2.01-5.74	0.159	2.04	0.76-5.50
hindfoot deformity	0.001	2.51	1.46-4.32	0.005	4.54	1.57-13.20
flat feet	0.249	1.35	0.81-2.25	-	-	-
myopia≥3D	<0.001	5.03	2.71-9.36	0.224	2.00	0.65-6.15
lens subluxation	<0.001	66.54	15.54-284.95	<0.001	67.12	6.51-691.63
excessive growth / remarkably high stature	<0.001	2.76	1.66-4.61	0.001	5.46	1.99-14.93
deficiency in weight	0.959	1.02	0.58-1.78	-	-	-
hernias	0.001	3.12	1.58-6.17	0.004	6.45	1.83-22.67
idiopathic pulmonary oedema	0.091	3.38	0.83-13.81	0.758	1.43	0.15-14.10

Table S1. Univariate and multivariate logistic analysies in order to establish independent predictors for the diagnosis of MFS in the entire population (children and adults)

Child population (n=178)	Univariate logistic regression			Multivariate logistic regression		
	p	HR	95%CI	p	HR	95%CI
USLS <0.85	0.984	0.98	0.19-5.07	-	-	-
ASHR >1.05	<0.001	6.84	2.99-15.64	0.003	11.48	2.29-57.48
thumb sign	0.097	2.00	0.89-4.54	0.888	1.13	0.21-6.03
wrist sign	0.088	1.84	0.91-3.69	0.875	0.88	0.18-4.36
pectus carinatum	0.027	2.57	1.11-5.95	0.808	0.80	0.13-4.94
pectus excavatum	0.329	1.44	0.70-2.96	-	-	-
scoliosis	0.175	1.81	0.77-4.25	-	-	-
assymetry of the chest	<0.001	8.62	2.91-25.51	0.119	4.33	0.69-27.38
joint laxity	0.922	1.04	0.48-2.24	-	-	-
joint hyperextension	0.621	0.84	0.41-1.71	-	-	-
reduced elbow extension	0.013	5.36	1.43-20.04	0.396	3.02	0.24-38.59
dolichocephaly	0.132	1.80	0.84-3.85	-	-	-
malar hypoplasia	0.801	1.09	0.55-2.20	-	-	-
enophthalmia	0.441	1.33	0.64-2.77	-	-	-
micrognation	0.008	2.61	1.28-5.31	0.545	0.58	1.00-3.39
retrognathia	0.074	1.97	0.94-4.13	0.919	0.91	0.15-5.63
downslanting palpebral fissures	0.001	3.58	1.74-7.39	0.152	2.71	0.69-10.58
gothic palate	<0.001	6.78	3.17-14.52	0.534	1.59	0.37-6.78
dental crowding	0.005	2.79	1.35-5.75	0.228	2.43	0.57-10.26
stretch marks	0.112	1.82	0.87-3.79	-	-	-
hindfoot deformity	<0.001	3.73	1.82-7.67	0.017	6.02	1.39-26.15
flat feet	0.139	1.77	0.83-3.76	-	-	-
myopia≥3D	0.002	4.02	1.68-9.59	0.732	0.67	0.07-6.59
lens subluxation	<0.001	94.29	11.93-745.00	0.001	78.91	5.51-1130.44
excessive growth / remarkably high stature	0.020	2.35	1.15-4.83	0.076	3.87	0.87-17.25
deficiency in weight	0.791	1.11	0.52-2.36	-	-	-
hernias	0.045	2.63	1.02-6.75	0.044	7.25	0.87-17.25
idiopathic pulmonary oedema	0.256	3.17	0.43-23.22	-	-	-

Table S2. Univariate and multivariate logistic analys~~i~~es in order to establish independent predictors for the diagnosis of MFS in the child population

Adult population (n=99)	Univariate logistic regression			Multivariate logistic regression		
	**p**	**HR**	**95%CI**	**p**	**HR**	**95%CI**
USLS <0.85	0.442	0.42	0.05-3.89	-	-	-
ASHR >1.05	0.099	2.51	0.84-7.51	0.810	0.73	0.06-9.13
thumb sign	0.002	4.18	1.71-10.22	0.269	3.78	0.36-40.07
wrist sign	0.080	2.17	0.91-5.19	0.622	0.56	0.05-5.74
pectus carinatum	0.001	6.64	2.25-19.56	0.131	4.75	0.63-35.79
pectus excavatum	0.420	1.66	0.48-5.71	-	-	-
scoliosis	0.277	1.89	0.60-5.95	-	-	-
assymetry of the chest	0.226	1.75	0.71-4.31	-	-	-
joint laxity	0.032	2.59	1.09-6.15	0.179	5.82	0.45-76.04
joint hyperextension	0.279	1.65	0.67-4.06	-	-	-
reduced elbow extension	0.037	1.74	1.43-3.27	0.999		
dolichocephaly	0.765	1.17	0.43-3.17	-	-	-
malar hypoplasia	0.410	1.43	0.61-3.33	-	-	-
enophthalmia	0.041	2.53	1.04-6.17	0.040	9.60	1.11-82.89
micrognation	0.558	0.78	0.33-1.82	-	-	-
retrognathia	0.770	1.15	0.45-2.97	-	-	-
downslanting palpebral fissures	0.010	3.60	1.35-9.57	0.807	0.77	1.00-6.23
gothic palate	<0.001	5.47	2.17-13.75	0.908	1.13	0.14-9.42
dental crowding	0.093	2.14	0.88-5.21	0.361	2.60	0.34-20.18
stretch marks	0.001	4.68	1.88-11.67	0.114	5.11	0.68-38.53
hindfoot deformity	0.013	13.68	1.73-108.24	0.119	10.61	0.55-206.61
flat feet	0.238	1.67	0.71-3.93	-	-	-
myopia≥3D	0.014	3.46	1.29-9.27	0.804	1.31	0.16-10.82
lens subluxation	0.001	30.11	3.82-237.45	0.998		
excessive growth / remarkably high stature	0.070	2.19	0.94-5.13	0.044	14.54	1.07-197.83
deficiency in weight	0.519	1.42	0.49-4.12	-	-	-
hernias	0.082	2.87	0.88-9.39	0.574	2.16	0.15-31.86
idiopathic pulmonary oedema	0.459	2.33	0.25-21.74	-	-	-

Table S3. Univariate and multivariate logistic analyseis in order to establish independent predictors for the diagnosis of MFS in the adult population

Feature	Adults (n=99)		
	**Women (n=54)**	**Men (n=45)**	**p**
age	31.5 ± 9.5	31.5 ± 9.5	0.548
BMI	22.1 ± 4.8	21.5 ± 4.6	0.536
body weight	67.2 ± 15.6	78.9 ± 15.9	**<0.001**
height	174.3 ± 9.6	191.6 ± 7.4	**<0.001**
ASHR >1.05	12 (22,2)	11 (24,4)	0.917
USLS < 0.85	47 (87,0)	41 (91,1)	1.000
joint laxity	37 (68,5)	15 (33,3)	**<0.001**
wrist sign	28 (51,9)	16 (35,6)	0.070
thumb sign	35 (64,8)	18 (40,0)	**0.006**
scoliosis	42 (77,8)	36 (80,0)	0.791
moderate or severe scoliosis	10 (18,5)	5 (11,1)	0.255
pectus excavatum	4 (7,4)	11 (24,4)	**0.024**
pectus carinatum	20 (37,0)	17 (37,8)	0.900
reduced elbow extension	3 (5,6)	4 (8,9)	0.700
flat feet	29 (53,7)	19 (42,2)	0.151
hindfoot deformity	9 (16,7)	9 (20,0)	0.680
stretch marks	25 (46,3)	24 (53,3)	0.576
dolichocephaly	15 (27,8)	13 (28,9)	0.868
dental crowding	25 (46,3)	16 (35,6)	0.295
gothic palate	24 (44,4)	27 (60,0)	0.153
downslanting palpebral fissures	24 (44,4)	11 (24,4)	**0.021**
retrognathia	12 (22,2)	14 (31,1)	0.391
micrognation	26 (48,1)	25 (55,6)	0.553
enophthalmia	25 (46,3)	35 (77,8)	**0.002**
malar hypoplasia	19 (35,2)	30 (66,7)	**0.002**
age at the time of first suspicion of MFS	20.3 ± 14.0	19.5 ± 13.2	0.780
birth weight	3092.1 ± 715.2	3795.0 ± 593.8	**0.049**
birth length	55.1 ± 4.8	56.6 ± 2.9	0.521
joint pain	39 (72,2)	15 (33,3)	**<0.001**
frequent headaches	37 (68,5)	15 (33,3)	**0.001**
dizziness	28 (51,9)	11 (24,4)	**0.007**
syncope	24 (44,4)	5 (11,1)	**<0.001**
chest pain	27 (50,0)	17 (37,8)	0.316
palpitations	23 (42,6)	9 (20,0)	**0.021**
coordination disorders	27 (50,0)	3 (6,7)	**<0.001**

hernias	6 (11,1)	15 (33,3)	**0.006**
idiopathic pulmonary oedema	3 (5,6)	2 (4,4)	1.000
multiple injuries	15 (27,8)	4 (8,9)	**0.018**
effort tolerance worse than that of peers	35 (64,8)	20 (44,4)	**0.039**
lens subluxation	16 (29,6)	12 (26,7)	0.946
myopia ≥ 3 D	23 (42,6)	14 (31,1)	0.565

Table S4. Comparative analysis of the most frequently reported symptoms, physical examination abnormalities and other more frequently noted abnormalities between women and men in the entire adult population.

**Feature**	**Adults with MFS (n=63)**		
	**Women with MFS (n=34)**	**Men with MFS (n=29)**	**p**
age	33.9 ± 14.2	32.8 ± 9.2	0.725
BMI	22.3 ± 5.1	21.7 ± 5.2	0.651
body weight	68.8 ± 16.1	80.1 ± 17.6	**0.011**
height	176.1 ± 9.0	192.2 ± 7.7	**<0.001**
ASHR >1.05	10 (29,4)	8 (27,6)	0.754
USLS < 0.85	29 (85,3)	26 (89,7)	0.617
joint laxity	26 (76,5)	12 (41,4)	**0.003**
wrist sign	19 (55,9)	13 (44,8)	0.388
thumb sign	26 (76,5)	15 (51,7)	**0.033**
scoliosis	28 (82,4)	23 (79,3)	0.694
moderate or severe scoliosis	9 (26,5)	3 (10,3)	0.093
pectus excavatum	4 (11,8)	7 (24,1)	0.187
pectus carinatum	16 (47,1)	16 (55,2)	0.477
reduced elbow extension	3 (8,8)	4 (13,8)	0.692
flat feet	21 (61,8)	12 (41,4)	0.074
hindfoot deformity	8 (23,5)	9 (31,0)	0.424
stretch marks	22 (64,7)	17 (58,6)	0.640
dolichocephaly	10 (29,4)	9 (31,0)	0.517
dental crowding	18 (52,9)	12 (41,4)	0.444
gothic palate	20 (58,8)	21 (72,4)	0.204
downslanting palpebral fissures	19 (55,9)	9 (31,0)	**0.034**
retrognathia	9 (26,5)	8 (27,6)	0.960

micrognation	16 (47,1)	15 (51,7)	0.670
enophthalmia	20 (58,8)	23 (79,3)	0.051
malar hypoplasia	12 (35,3)	21 (72,4)	**0.002**
age at the time of first suspicion of MFS	17.7 ± 14.7	18.0 ± 14.1	0.950
birth weight	3168.8 ± 735.3	3966.7 ± 450.9	0.118
birth length	53.5 ± 4.4	58.5 ± 3.5	0.178
joint pain	23 (67,6)	10 (34,5)	**0.016**
frequent headaches	22 (64,7)	9 (31,0)	**0.014**
dizziness	19 (55,9)	9 (31,0)	0.061
syncope	17 (50,0)	4 (12,8)	**0.004**
chest pain	17 (50,0)	12 (41,4)	0.683
palpitations	16 (47,1)	6 (20,7)	**0.036**
coordination disorders	17 (50,0)	3 (10,3)	**0.001**
hernias	6 (17,6)	11 (37,9)	0.054
idiopathic pulmonary oedema	2 (5,9)	2 (6,9)	1.000
multiple injuries	10 (29,4)	1 (3,4)	**0.016**
effort tolerance worse than that of peers	23 (67,6)	12 (41,4)	**0.030**
lens subluxation	15 (44,1)	12 (41,4)	0.875
myopia ≥ 3 D	17 (50,0)	12 (41,4)	0.973

Table S5. Comparative analysis of the most frequently reported symptoms, physical examination abnormalities and other more frequently noted abnormalities between women and men in the population of adults with Marfan syndrome.

**Feature**	**Adults with marfanoid habitus (n=36)**		
	**Women (n=20)**	**Men (n=16)**	**p**
age	31.2 ± 8.3	29.2 ± 10.0	0.525
BMI	21.9 ± 4.4	21.2 ± 3.5	0.646
body weight	64.3 ± 14.7	76.6 ± 12.1	**0.014**
height	171.3 ± 10.0	190.4 ± 6.8	**<0.001**
ASHR >1.05	2	3	0.642
USLS < 0.85	18	15	0.471
joint laxity	11	3	**0.012**
wrist sign	9	3	0.057
thumb sign	9	3	0.080

scoliosis	14	13	1.000
moderate or severe scoliosis	1	2	0.591
pectus excavatum	0	4	**0.044**
pectus carinatum	4	1	0.335
reduced elbow extension	0	0	-
flat feet	8	7	0.968
hindfoot deformity	1	0	1.000
stretch marks	3	7	0.134
dolichocephaly	5	4	0.688
dental crowding	7	4	0.458
gothic palate	4	6	0.457
downslanting palpebral fissures	5	2	0.405
retrognathia	3	6	0.250
micrognation	10	10	0.738
enophthalmia	5	12	**0.015**
malar hypoplasia	7	9	0.492
age at the time of first suspicion of MFS	24.5 ± 12.1	22.4 ± 11.2	0.616
birth weight	2990.0 ± 742.4	3623.3. ± 767.9	0.271
birth length	58.3 ± 4.3	55.3 ± 2.1	0.333
joint pain	16	5	**0.002**
frequent headaches	15	6	**0.002**
dizziness	9	2	0.064
syncope	7	1	0.053
chest pain	10	5	0.260
palpitations	7	3	0.451
coordination disorders	10	0	**0.001**
hernias	0	4	**0.033**
idiopathic pulmonary oedema	1	0	1.000
multiple injuries	5	3	0.699
effort tolerance worse than that of peers	12	8	0.563
lens subluxation	1	0	1.000
myopia ≥ 3 D	6	2	0.408

Table S6. Comparative analysis of the most frequently reported symptoms, physical examination abnormalities and other more frequently noted abnormalities between women and men in the population of adults with marfanoid habitus.

**Feature**	**Adult men (n=45)**		
	**MFS (n=29)**	**Marfanoid habitus (n=16)**	**p**
age	32.8 ± 9.2	29.2 ± 10.0	0.228
BMI	21.7 ± 5.2	21.2 ± 3.5	0.750
body weight	80.1 ± 17.6	76.6 ± 12.1	0.494
height	192.2 ± 7.7	190.4 ± 6.8	0.422
deficiency in weight	6 (20,7)	3 (18,8)	1.000
remarkably high stature	18 (62,1)	8 (50,0)	0.739
ASHR >1.05	8 (27,6)	3 (18,8)	0.720
USLS < 0.85	26 (89,7)	15 (93,8)	1.000
joint laxity	12 (41,4)	3 (18,8)	0.087
wrist sign	13 (44,8)	3 (18,8)	0.054
thumb sign	15 (51,7)	3 (18,8)	**0.018**
scoliosis	23 (79,3)	13 (81,3)	1.000
moderate or severe scoliosis	3 (10,3)	2 (12,5)	1.000
pectus excavatum	7 (24,1)	4 (25,0)	1.000
pectus carinatum	16 (55,2)	1 (6,25)	**0.001**
reduced elbow extension	4 (13,8)	0	0.279
flat feet	12 (41,4)	7 (43,8)	0.965
hindfoot deformity	9 (31,0)	0	**0.008**
stretch marks	17 (58,6)	7 (43,8)	0.220
dolichocephaly	9 (31,0)	4 (25,0)	0.414
dental crowding	12 (41,4)	4 (25,0)	0.218
gothic palate	21 (72,4)	6 (37,5)	**0.008**
downslanting palpebral fissures	9 (31,0)	2 (12,5)	0.166
retrognathia	8 (27,6)	6 (37,5)	0.594
micrognation	15 (51,7)	10 (62,5)	0.655
enophthalmia	23 (79,3)	12 (75,0)	0.443
malar hypoplasia	21 (72,4)	8 (50,0)	0.137
age at the time of first suspicion of MFS	18.0 ± 14.1	22.4 ± 11.2	0.309
birth weight	3966.7 ± 450.9	3623.3 ± 767.9	0.541
birth length	58.5 ± 3.5	55.3 ± 2.1	0.283
joint pain	10 (34,5)	5 (31,3)	0.743
frequent headaches	9 (31,0)	6 (37,5)	0.730
dizziness	9 (31,0)	2 (12,5)	0.168
syncope	4 (13,8)	1 (6,25)	0.636
chest pain	12 (41,4)	5 (31,3)	0.422
palpitations	6 (20,7)	3 (18,8)	1.000

coordination disorders	3 (10,3)	0	0.287
hernias	11 (37,9)	4 (25,0)	0.362
idiopathic pulmonary oedema	2 (6,9)	0	0.530
multiple injuries	1 (3,4)	3 (18,8)	0.130
effort tolerance worse than that of peers	12 (41,4)	8 (50,0)	0.658
lens subluxation	12 (41,4)	0	**0.002**
myopia ≥ 3 D	12 (41,4)	2 (12,5)	**0.024**

Table S7. Comparative analysis of the most frequently reported symptoms, physical examination abnormalities and other more frequently noted abnormalities between patients with Marfan syndrome and marfanoid habitus in the group of adult men.

**Feature**	**Adult women (n=54)**		
	**MFS (n=34)**	**Marfanoid habitus (n=20)**	**p**
age	33.9 ± 14.2	31.2 ± 8.3	0.438
BMI	22.3 ± 5.1	21.9 ± 4.4	0.758
body weight	68.8 ± 16.1	64.3 ± 14.7	0.321
height	176.1 ± 9.0	171.3 ± 10.0	0.086
deficiency in weight	8 (23,5)	3 (15,0)	0.505
remarkably high stature	19 (55,9)	5 (25,0)	**0.029**
ASHR >1.05	10 (29,4)	2 (10,0)	0.171
USLS < 0.85	29 (85,3)	18 (90,0)	0.544
joint laxity	26 (76,5)	11 (55,0)	0.180
wrist sign	19 (55,9)	9 (45,0)	0.522
thumb sign	26 (76,5)	9 (45,0)	**0.021**
scoliosis	28 (82,4)	14 (70,0)	0.398
moderate or severe scoliosis	9 (26,5)	1 (5,0)	0.070
pectus excavatum	4 (11,8)	0	0.284
pectus carinatum	16 (47,1)	4 (20,0)	0.073
reduced elbow extension	3 (8,8)	0	0.544
flat feet	21 (61,8)	8 (40,0)	0.110
hindfoot deformity	8 (23,5)	1 (5,0)	0.130
stretch marks	22 (64,7)	3 (15,0)	**<0.001**
dolichocephaly	10 (29,4)	5 (25,0)	0.736
dental crowding	18 (52,9)	7 (35,0)	0.239
gothic palate	20 (58,8)	4 (20,0)	**0.006**

downslanting palpebral fissures	19 (55,9)	5 (25,0)	**0.024**
retrognathia	9 (26,5)	3 (15,0)	0.494
micrognation	16 (47,1)	10 (50,0)	0.706
enophthalmia	20 (58,8)	5 (25,0)	**0.027**
malar hypoplasia	12 (35,3)	7 (35,0)	0.923
age at the time of first suspicion of MFS	17.7 ± 14.7	24.5 ± 12.1	0.075
birth weight	3168.8 ± 735.3	2990.0 ± 742.4	0.662
birth length	53.5 ± 4.4	58.3 ± 4.3	0.105
joint pain	23 (67,6)	16 (80,0)	0.328
frequent headaches	22 (64,7)	15 (75,0)	0.347
dizziness	19 (55,9)	9 (45,0)	0.405
syncope	17 (50,0)	7 (35,0)	0.307
chest pain	17 (50,0)	10 (50,0)	0.938
palpitations	16 (47,1)	7 (35,0)	0.361
coordination disorders	17 (50,0)	10 (50,0)	0.869
hernias	6 (17,6)	0	0.078
idiopathic pulmonary oedema	2 (5,9)	1 (5,0)	1.000
multiple injuries	10 (29,4)	5 (25,0)	0.797
effort tolerance worse than that of peers	23 (67,6)	12 (60,0)	0.409
lens subluxation	15 (44,1)	1 (5,0)	**0.002**
myopia ≥ 3 D	17 (50,0)	6 (30,0)	0.159

Table S8. Comparative analysis of the most frequently reported symptoms, physical examination abnormalities and other more frequently noted abnormalities between patients with Marfan syndrome and marfanoid habitus in the group of adult women.
